# Oracle Upper Bounds on Clean-EEG Recoverability from Single-Channel Decompositions Under EOG/EMG Contamination

**DOI:** 10.3390/s26092581

**Published:** 2026-04-22

**Authors:** Usman Qamar Shaikh, Anubha Manju Kalra, Andrew Lowe, Imran Khan Niazi

**Affiliations:** 1 Institute of Biomedical Technologies, Auckland University of Technology, Auckland 1010, New Zealand; anubha.kalra@aut.ac.nz (A.M.K.); andrew.lowe@aut.ac.nz (A.L.); 2 Department of Electrical and Electronic Engineering, School of Engineering Computer and Mathematical Sciences, Auckland University of Technology, Auckland 1010, New Zealand; 3 Department of Mechanical Engineering, School of Engineering Computer and Mathematical Sciences, Auckland University of Technology, Auckland 1010, New Zealand; 4 Centre for Chiropractic Research, New Zealand College of Chiropractic, Auckland 1060, New Zealand; imran.niazi@nzchiro.co.nz; 5 Department of Health Science and Technology, Aalborg University, 9200 Aalborg, Denmark

**Keywords:** electroencephalography (EEG), artifact suppression, ocular artifacts (EOG), muscle artifacts (EMG), signal decomposition, variational mode decomposition (VMD), CEEMDAN, singular spectrum analysis (SSA), wavelet transform (DWT)

## Abstract

Objective: Single-channel EEG artifact suppression often relies on signal decomposition; however, it is not always clear how much clean EEG is recoverable from a given decomposition when component weighting is ideal. We present an oracle-based benchmark that characterises this best-case recoverability across common 1-D decomposition families under controlled EOG, EMG, and mixed contamination. This work does not propose a new denoising algorithm; rather, it isolates representation capacity from component-selection heuristics by computing an upper bound on reconstruction quality. Approach: Using EEGdenoiseNet, we constructed a synthetic benchmark of 4500 single-channel 2 s segments (125 Hz; T = 250) by mixing clean EEG with ocular (EOG) and/or cranial EMG exemplars at noise-to-signal ratios (NSRs) spanning −10 to +10 dB (floor −10 dB denotes an absent modality). We evaluated variational mode decomposition (VMD), singular spectrum analysis (SSA), discrete wavelet transform (DWT), and CEEMDAN by decomposing each mixture and reconstructing the clean EEG using a bounded nonnegative linear combination of components obtained via constrained least squares (the oracle). Main results: Under this oracle benchmark, SSA achieved the lowest reconstruction error in most tested conditions, while DWT tended to rank best in milder ocular regimes; VMD performance improved, with an increased mode count at higher computational cost. CEEMDAN exhibited higher latency dominated by ensemble settings. Significance: These results should be interpreted as decomposition-level upper bounds under controlled mixtures, not field-ready denoising performance. The benchmark provides a tool with which to compare representational recoverability across decompositions and to inform the subsequent design of practical component-selection strategies.

## 1. Introduction

Electroencephalography (EEG) is a widely used non-invasive technique for monitoring brain activity with millisecond temporal resolution and comparatively low-cost instrumentation, supporting applications ranging from clinical assessment to brain–computer interfaces and the longitudinal monitoring of cognitive states [1,2,3,4,5,6,7,8]. In parallel, there is growing interest in wearable and limited-lead EEG (e.g., headbands, ear-EEG, and EEG integrated into mobile or VR form factors) to improve comfort and support use outside the laboratory [9,10,11,12,13]. These designs can, however, make analysis more sensitive to non-neural contamination, particularly when fewer channels are available [14].

EEG is low-amplitude (tens of microvolts) and is, therefore, susceptible to physiological artifacts, especially ocular activity (EOG) from blinks and eye movements and muscle activity (EMG) from cranial and facial muscles [15,16,17]. These contaminants can reach amplitudes comparable to, or larger than, the underlying neural signal and can overlap with conventional EEG bands (ocular transients often dominate low frequencies, while EMG contributes broadband activity extending into higher frequencies). Such overlap can distort event-related potentials, and bias spectral or connectivity measures, with effects that may be more pronounced in ambulatory settings, where blinking and facial activity are frequent [17,18,19].

A broad toolbox exists for artifact mitigation in multichannel EEG; however, many common approaches rely on spatial redundancy, which is not present in single-lead recordings. Blind source separation and subspace methods (e.g., independent component analysis (ICA) and artifact subspace reconstruction (ASR)) typically exploit cross-channel structures to separate artifact-dominated sources from neural activity [20,21,22,23], while regression methods that use auxiliary reference channels may be impractical in some wearable deployments [24,25,26,27]. As a result, single-channel pipelines often turn to time–frequency or data-adaptive decompositions that map a 1-D mixture into multiple components that can be selectively attenuated, including wavelet transforms, empirical mode decomposition (EMD) family methods, variational mode decomposition (VMD), and singular spectrum analysis (SSA).

Wavelet decompositions, including discrete wavelet transform (DWT) and stationary wavelet transform (SWT), are widely used for single-channel EEG denoising because they provide a multiscale representation (approximation plus detail sub-bands) that can support sub-band selection and/or coefficient thresholding during reconstruction [28,29,30,31,32]. Their behaviour is shaped by a small set of practical hyperparameters, most notably the mother wavelet, decomposition level, and thresholding/shrinkage rule (global vs. band-wise); published results suggest that these choices can be condition- and dataset-dependent. For instance, Khatun et al. examined blink removal under multiple wavelets and thresholding schemes and reported sensitivity to these design choices [32].

EMD-family methods are widely used for single-channel EEG denoising because they provide a data-adaptive decomposition of the signal into intrinsic mode functions (IMFs) plus a residual, often yielding components ordered from faster to slower oscillations without assuming stationarity [33]. To reduce the mode-mixing seen in classical EMD, noise-assisted variants such as ensemble empirical mode decomposition (EEMD) and complete ensemble empirical mode decomposition with adaptive noise (CEEMDAN) perform ensemble decompositions with added noise, with CEEMDAN commonly used when more stable IMFs are desired [34,35]. In practice, CEEMDAN’s behaviour is influenced by a few key hyperparameters, the added noise amplitude, the ensemble size, and the maximum number of IMFs, which can trade reconstruction quality against runtime and may vary with artifact regimes [36]. Recent studies illustrate both the popularity of these methods and the variability in evaluation choices (e.g., comparisons across EMD/EEMD/CEEMDAN on EMG/ECG denoising [37], and bidimensional empirical mode decomposition-based ERP denoising compared with EEMD-based baselines [38]).

Variational mode decomposition (VMD) is a commonly used single-channel decomposition method that represents a signal as a sum of K band-limited modes, each centred around an adaptively estimated frequency via an optimisation procedure [39]. In EEG denoising, VMD is often used to generate candidate components that can be attenuated or selected during reconstruction without requiring multichannel spatial information [40]. Its practical behaviour is influenced by a small set of hyperparameters, most notably, the number of modes (K), which control decomposition granularity and can affect both separability and runtime; other settings (e.g., penalty/bandwidth and stopping criteria) are frequently kept at defaults in applied work. Recent single-channel studies have used VMD mainly for ocular artifact suppression with fixed or heuristically chosen settings, e.g., two-stage VMD pipelines for blink removal [41], Multiscale modified sample entropy-guided VMD component regression [42], and hybrid VMD–BSS approaches reporting dataset-specific preferences for K [40].

Furthermore, recent work has expanded VMD-based denoising through hybrid selector stages that explicitly identify artifact-related modes or localise artifacts in the time–frequency domain. For example, Kaur et al. proposed a hybrid VMD–detrended fluctuation analysis (DFA)–wavelet approach in which VMD modes are screened using DFA/Hurst-type criteria and only the flagged modes are further denoised using wavelet transforms, with evaluation reported on simulated mixtures and a real depression dataset [43]. Other recent studies have also explored VMD-family variants and hybrid frameworks for artifact removal, including successive or adaptive VMD strategies coupled with automated component identification, and muscle-artifact pipelines combining VMD with wavelet-domain localisation and correlation-based separation [44,45]. Collectively, this literature reinforces that VMD’s realised denoising performance depends not only on the decomposition itself (notably K) but also on the downstream mode-selection or weighting rule, motivating decomposition-centric evaluation under ideal weighting and systematic hyperparameter sweeps, as performed in this study.

Singular spectrum analysis (SSA) is a single-channel decomposition method that embeds a time series into a trajectory (Hankel) matrix and uses singular value decomposition to reconstruct a set of components that can be recombined for denoising [46,47]. In EEG applications, SSA is often used as a data-adaptive component generator followed by heuristic selection or attenuation of components before reconstruction [48]. Its behaviour is largely governed by the window length (L) and the associated grouping/retention strategy, which influence both separability and the number of components to manage [48]. Prior work has applied SSA to ocular blink suppression with clustering-based selection [49], to EMG attenuation using mobility-type criteria [48], and to multi-artifact settings when combined with ICA (e.g., SSA–SOBI) under fixed design choices and semi-simulated mixtures [50].

In parallel with the decomposition-based denoising, recent work has increasingly explored end-to-end deep learning for single-channel EEG artifact removal, including convolutional neural network (CNN)–transformer hybrids, decision-guided routing networks, and transformer-based denoisers that fuse local and non-local structure [51,52,53,54]. These models typically learn a direct nonlinear mapping from contaminated to clean EEG using semi-simulated mixtures (e.g., EEGdenoiseNet) and report signal-level metrics on those benchmarks. However, their operation is usually “always-on”; the learned mapping is less transparent than decomposition pipelines that expose intermediate components and provide explicit handles (e.g., number of modes, wavelet family/level, window length) for controlling latency and behaviour. Accordingly, deep denoisers and decomposition methods address complementary needs: learned models can provide powerful nonlinear suppression, whereas decomposition pipelines can be more interpretable and tuneable, but are sensitive to hyperparameter choice and component-selection rules.

Across decomposition-based approaches, several practical considerations recur. Performance depends on design choices (e.g., decomposition depth, basis or penalty settings, ensemble size) that influence component separability, computational cost, and downstream selection burden, and these settings are often chosen heuristically. Moreover, end-to-end denoising outcomes conflate the capabilities of the decomposition itself with those of the downstream selector. To disentangle these factors, we quantify decomposition recoverability capacity under ideal component weighting via an oracle reconstruction, providing an upper-bound reference and stability/latency trade-offs that can guide practical selector design and controlled deployment.

Recent denoising studies, including both decomposition-based hybrids and end-to-end deep networks, are typically evaluated as complete pipelines in which the decomposition, selector, and reconstruction stages are intertwined. As a result, it is often unclear whether observed performance differences arise from the representational capacity of the decomposition itself or from the downstream selection rule. The present work addresses this gap by introducing an oracle-based benchmark that isolates decomposition recoverability under ideal component weighting, while also characterising hyperparameter stability and performance–latency trade-offs across method families.

These considerations motivate several practical questions:

- RQ1: Are decomposition hyperparameters (e.g., depth/ensemble size/mode count) reasonably stable across artifact types and contamination strengths, or do preferred settings shift by regime?
- RQ2: Do different decomposition families show regime-dependent advantages (EOG, EMG, mixed; low vs. high NSR), or is any one family consistently competitive?
- RQ3: How does decomposition depth affect the performance–runtime balance, and where do diminishing returns appear?

Here, we use an oracle-based benchmarking approach to examine the representational behaviour of several common single-channel decomposition families under controlled conditions. Using a benchmark with a known clean reference EEG, we sweep hyperparameter grids for DWT, CEEMDAN, VMD, and SSA. For each noisy epoch, we compute an oracle reconstruction as a bounded, nonnegative linear recombination of the decomposition components, which serves as a reference point for what can be recovered, given that representation. We summarise results by artifact kind (EOG, EMG, mixed) and by noise-to-signal ratio (NSR) bins, rather than relying only on pooled averages. To relate oracle results to more practical settings, we also report heuristic operating points: Best, an effect-size–aware Opt1 rule that selects the fastest configuration that is negligibly different from the bin-wise Best, and a Pareto-based Opt2 criterion to summarise performance–latency trade-offs under consistent tuning rules.

In this work, we:

- evaluate DWT, CEEMDAN, VMD, and SSA under controlled hyperparameter sweeps in a single-channel benchmark;
- compute oracle reconstructions to summarise what is recoverable from each decomposition independent of a specific selector;
- report performance by artifact kind (EOG, EMG, mixed) and by NSR bins, rather than only pooled averages;
- summarise practical settings using Best/Opt1/Opt2 criteria to reflect performance–latency trade-offs.

This study is intentionally framed as a recoverability benchmark rather than a deployable artifact-removal pipeline. By using an oracle that has access to the clean target, we quantify an upper bound on what each decomposition family can reconstruct when component weighting is ideal, independent of any practical component-selection rule. Consequently, conclusions are restricted to the controlled benchmark conditions and are intended to guide subsequent development and evaluation of implementable selectors.

The remainder of the paper describes the benchmark construction and decomposition grids, defines the constrained oracle reconstruction and evaluation metrics, introduces Best/Opt1/Opt2 selection, and reports within-method hyperparameter behaviour and inter-method comparisons stratified by artifact kind and NSR.

## 2. Methods

### 2.1. The Benchmark Dataset and Noise Synthesis

We constructed a synthetic single-channel EEG benchmark by mixing clean EEG segments with EOG and cranial EMG artifact exemplars under controlled noise-to-signal ratios (NSRs). Clean EEG, EOG, and EMG epochs were taken from EEGdenoiseNet open dataset [55], treating each channel as an independent 2 s epoch. All epochs were resampled from 256 Hz to 125 Hz, yielding segments of length T = 250 samples.

For this decomposition benchmark we generated 4500 synthetic 2 s segments with paired clean references. Each synthetic segment Y was generated by selecting a clean EEG epoch X and (optionally) an EOG epoch $N_{EOG}$ and/or an EMG epoch $N_{EMG}$, then forming a linear mixture, as given in Equation (1):

(1) $$\begin{array}{cccc} & Y\;\;=X+\lambda_{EOG}N_{EOG}+\lambda_{EMG}N_{EMG}. & & \end{array}$$

The gains $\lambda_{EOG}$ and $\lambda_{EMG}$ were chosen to achieve desired EOG and EMG NSRs relative to the clean EEG power. For a given 2 s segment, the EOG and EMG NSRs (in dB) are given in Equations (2) and (3):

(2) $$\begin{array}{cccc} & Z_{EOG}=10{log}_{10}\left(\lambda_{EOG}^{2}\frac{mean(N_{EOG}^{2})}{mean(X^{2})}\right), & & \end{array}$$

(3) $$\begin{array}{cccc} & Z_{EMG}=10{log}_{10}\left(\lambda_{EMG}^{2}\frac{mean(N_{EMG}^{2})}{mean(X^{2})}\right). & & \end{array}$$

Target NSR values were sampled uniformly from [−10,10] dB, spanning nearly clean (−10 dB) to heavily contaminated (+10 dB) conditions. A floor value of −10 dB was used to represent an “absent” artifact modality in single-artifact segments.

We generated three mixture types with fixed proportions:

- EOG-only (N = 1250): $Z_{EOG}∼U[-10,10]$ and $Z_{EMG}$ fixed at the floor;
- EMG-only (N = 1250): $Z_{EMG}∼U[-10,10]$ and $Z_{EOG}$ fixed at the floor;
- Mixed EOG + EMG (N = 2000): a total NSR $Z_{tot}∼U[-10,10]$ was drawn and split between EOG and EMG power via a random EOG share r∈[0,1]. Let $q_{tot}\;=\;10^{Z_{tot}/10}$. And define $q_{EOG}\;=\;r\;q_{tot}$ and $q_{EMG}\;=\;(1-r)\;q_{tot}$. The share r was sampled using three patterns (uniform, increasing with $Z_{tot}$, decreasing with $Z_{tot}$) to obtain EMG-dominant, EOG-dominant, and balanced mixtures across NSR. Table 1 reports the number of epochs in each NSR bin for the EOG-only, EMG-only, and mixed subsets.

**Figure 1:**
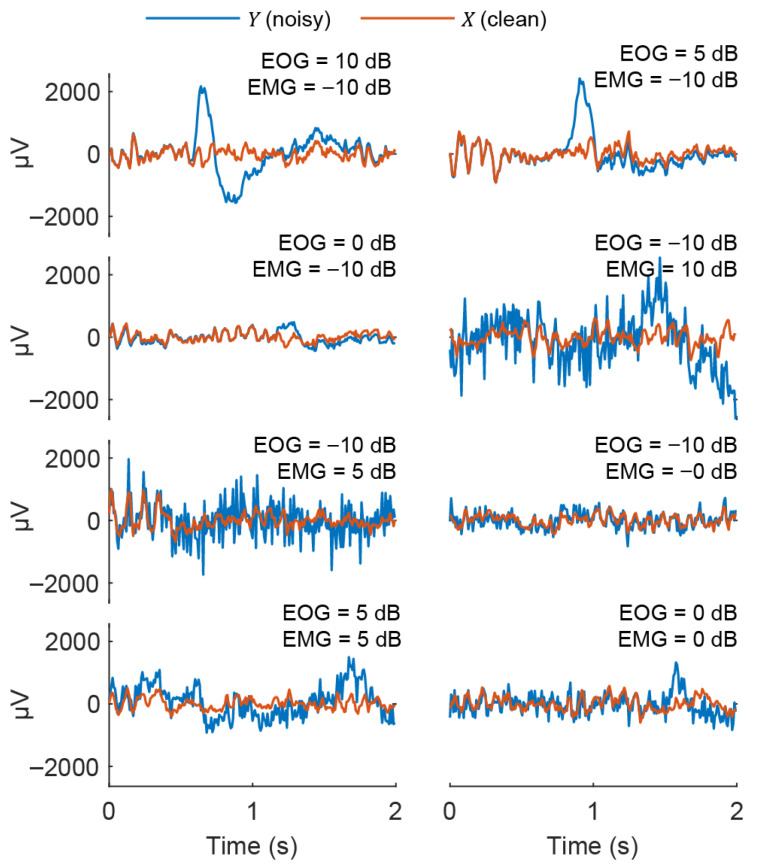
Example of synthetic samples for benchmarking. Each panel shows a 2 s noisy sample Y (blue) overlaid with its clean EEG source X (orange) for different combinations of EOG and EMG artifact loads in dB.

For each synthetic segment we obtained: the noisy observation Y; the clean EEG X; the scaled artifact components $N_{EOG}^{*}$ and $N_{EMG}^{*}$; continuous NSR labels $\left(Z_{EOG}|Z_{EMG}\right)$; and a categorical label kind∈{EOG,emg,mixed}. All methods observed the same 4500 noisy segments Y  and were evaluated on how well an optimal combination of their decomposition components can recover the ground-truth EEG X. Some examples of synthetic sample pairs are shown in Figure 1.

### 2.2. Decomposition Methods and Hyperparameter Grids

We benchmarked four one-dimensional decomposition families commonly used for EEG denoising and time–frequency analysis: VMD, SSA, DWT, and CEEMDAN. All methods were applied to the same 2 s single-channel segments Y (125 Hz, T = 250). For every method and hyperparameter configuration, the decomposition can be written as:

(4) $$\begin{array}{cccc} & Y\left(t\right)=\;\sum_{k\;=\;1}^{K}c_{k}(t), & & \end{array}$$

where $c_{k}(t)$ are modes, components, IMFs, or sub-bands.

#### 2.2.1. Variational Mode Decomposition (VMD)

VMD decomposes a signal into K band-limited modes [39]. We used the MATLAB R2023b implementation (Signal Processing Toolbox), keeping regularisation and stopping criteria at their default values and sweeping only the number of modes:

K∈{4,5,6,…,24}.

This yields a total of 21 VMD configurations.

#### 2.2.2. Singular Spectrum Analysis (SSA)

SSA is a linear, data-adaptive decomposition based on Hankel embedding followed by SVD [46]. We used the MATLAB R2023b implementation and treated each rank-1 reconstructed component as a separate mode. The primary hyperparameter is the embedding window length L (samples), swept as:

L∈{8,16,24,…,248}.

This yielded a total of 31 SSA configurations.

#### 2.2.3. Discrete Wavelet Transform (DWT)

DWT provides a multiresolution filter-bank decomposition defined by a mother wavelet and decomposition level [28]. We used MATLAB R2023b’s standard DWT implementation. For each (wName, J) pair, we computed the DWT and treated the approximation and all detail sub-bands as components. Each sub-band was mapped back to a length-T time series via inverse reconstruction of that sub-band alone (other coefficients set to zero), and these reconstructed sub-band signals were used as components in the oracle recombination. The grid was as follows:

- wName∈{sym4,db4,coif3};
- J∈{4,5,6,7,8,9,10,12,14,16}.

This yielded a total of 30 DWT configurations.

#### 2.2.4. CEEMDAN

Complete ensemble empirical mode decomposition with adaptive noise (CEEMDAN) is a noise-assisted extension of EMD that extracts intrinsic mode functions (IMFs), while averaging across noise realisations [35]. We used the original CEEMDAN MATLAB implementation released by the authors [56] and swept across three hyperparameters:

- $N_{std}\in \{0.1,0.2,0.3\}$;
- $N_{R}\in \{30,50,100\}$;
-
MaxIMF∈{8,12,16}.

This yielded a total of 27 CEEMDAN configurations. All extracted IMFs (up to MaxIMF) were treated as components. Any residual beyond the last IMF was included as an additional component.

### 2.3. Oracle Reconstruction and Performance Metrics

For a noisy segment $Y\in R^{T}$, each decomposition produces K components $\{c_{k}{\}}_{k\;=\;1}^{K}$, where K depends on the method and hyperparameter setting. We stacked the components as follows:

(5) $$C\;=\;\left[\begin{array}{c} c_{1}^{⊤}\\ \vdots \\ c_{K}^{⊤} \end{array}\right]\in R^{K\times T}.$$

We defined an oracle linear reconstructor as follows:

(6) $$\begin{array}{cccc} & \hat{X}\left(t\right)=\sum_{k\;=1}^{K}w_{k}\;c_{k}(t), & & \end{array}$$

with weights $w=\;[w_{1},\ldots ,w_{K}{]}^{T}$, chosen to minimise the reconstruction error under bounded, nonnegative mixing:

(7) $$\begin{array}{cccc} & \underset{w}{min}{\left\|X-C^{T}w\right\|}_{2}^{2}\;s.t.\;0\leq w_{k}\leq 1\|\forall k. & & \end{array}$$

The box constraints limit sign inversions and large cancellations, making the oracle behave like a soft component-selection/attenuation mechanism. The constrained least-squares problem was solved using MATLAB lsqlin function.

Per segment, the relative root mean square error (RRMSE) and Pearson correlation coefficient (PCC), are given in Equations (8) and (9):

(8) $$RRMSE\;=\frac{{\left\|X-\hat{X}\right\|}_{2}}{{\left\|X\right\|}_{2}}$$

(9) $$PCC=corr(X,\hat{X})$$

**Figure 2:**
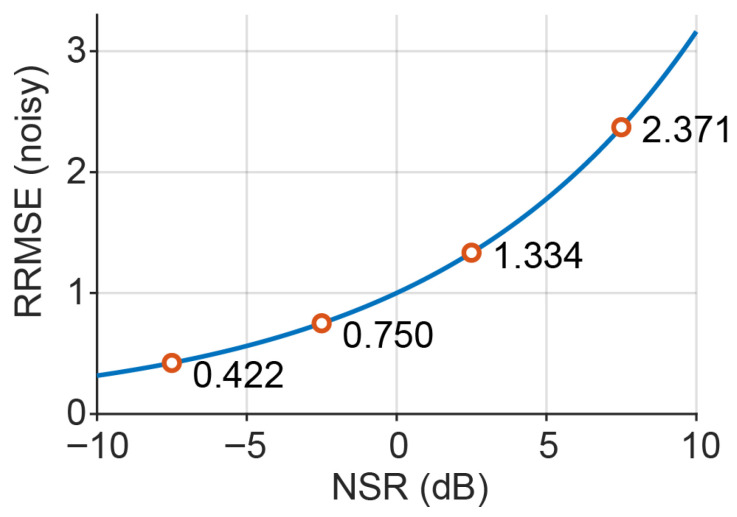
Analytical mapping between noise-to-signal ratio (NSR, dB) and relative RMSE (RRMSE).

**Figure 3:**
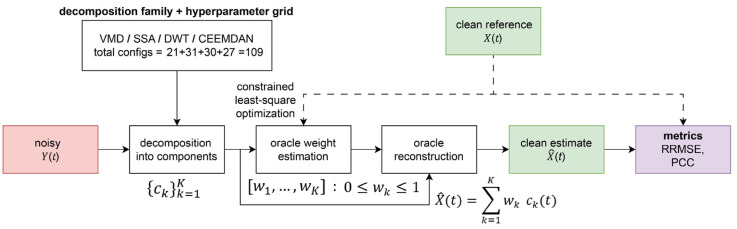
Oracle benchmarking pipeline.

The analytical relationship between NSR (dB) and the corresponding unprocessed baseline error (RRMSE of noisy) is illustrated in Figure 2. Figure 3 summarises the oracle benchmarking pipeline.

#### Practical Selector Baseline (Wavelet Shrinkage)

To provide a minimal, deployable reference point alongside the oracle benchmark, we additionally evaluated a practical selector within the wavelet family, where established coefficient-thresholding rules exist. Using the same validation subset, we applied discrete wavelet decomposition (DWT) with a fixed depth L = 6 and compared three commonly used mother wavelets (sym4, db4, coif3). For each epoch, wavelet coefficients were soft-thresholded using the universal threshold (UT) rule, applied to detail sub-bands at levels 1–6. The inverse transform yielded a thresholded reconstruction consistent with our implementation. Performance was quantified using the same metrics as the oracle benchmark (RRMSE and PCC). To assess whether differences between mother wavelets were robust under this practical baseline, we performed paired Wilcoxon signed-rank tests on per-epoch RRMSE with Holm correction applied within each NSR bin across the three pairwise wavelet comparisons; we additionally report the median paired difference and win rate (fraction of epochs where one wavelet yields lower RRMSE than the other).

### 2.4. Hyperparameter Selection: Best, Opt1, and Opt2

For each method, contamination kind, and NSR bin, we evaluated a discrete hyperparameter grid H. For each configuration h∈H, we computed: (i) mean oracle reconstruction error $\bar{RRMSE}(h)$ across epochs in that stratum; and (ii) mean decomposition time ${\bar{t}}_{dec}(h)$.

We stratified the benchmark by:

- Kind: EOG-only, EMG-only, and mixed;
- NSR axis: $Z_{EOG}$ for EOG, $Z_{EMG}\;$ for EMG, and $Z_{tot}\;$ for mixed;
- Bins: [−10,−5),[−5,0),[0,5),[5,10], plus the pooled range ALL [−10,10].

#### 2.4.1. Best

We define the performance–optimal configuration as:

(10) $$h_{Best}^{*}\;=\;arg\;\underset{h\in H}{min}\;\bar{RRMSE}(h),$$

i.e., the configuration with the lowest mean RRMSE in that (method, kind, bin), ignoring runtime.

#### 2.4.2. Opt1 (Effect-Size–Aware)

To identify configurations that are practically indistinguishable from Best, we computed Cohen’s d for each h relative to Best within the same stratum:

(11) $$d\left(h\right)=\;\frac{\mu_{h}-\mu_{Best}}{s_{pooled}},$$

where $\mu_{h}$ and $\mu_{Best}\;$ are the mean RRMSE across epochs for configuration h and for $h_{Best}^{*}$, respectively, and $s_{pooled}\;$ is the pooled standard deviation across the two per-epoch RRMSE distributions. We define the tolerance set as follows:

(12) $$H_{tol}\;=\;\left\{h\in H:\left|d\left(h\right)\right|\leq d_{0}\right\},{\;\;\;\;\;\;\;\;\;\;d}_{0}\;=0.05,$$

and select the fastest member as:

(13) $$h_{Opt1}^{*}\;=\;arg\;\underset{h\in H_{tol}}{min}{\bar{t}}_{dec}(h).$$

We used a deliberately stringent threshold ($d_{0}\;=\;0.05$) to operationalise visually/clinically negligible differences while prioritising speed.

#### 2.4.3. Opt2 (Pareto + Utopia)

Opt2 performs a two-objective selection over $\left(\bar{RRMSE}(h),{\bar{t}}_{dec}(h)\right)$. We first identified the Pareto-optimal set, then rescaled both objectives to [0,1] over the Pareto points (min–max scaling), and chose the point closest to the utopia point (0,0) in the rescaled space.

Opt1 and Opt2 optimise different notions of practicality. Opt1 explicitly optimises for speed subject to a near-Best performance constraint ($\left|d\right|\leq d_{0}$), and, therefore, tends to select the fastest configuration among those that appear effectively tied with Best. Opt2 treats performance and latency as co-objectives, identifies non-dominated (Pareto-optimal) settings, and selects a balanced point. As a result, Opt2 can accept a larger performance drop than Opt1 if it yields a meaningful latency reduction.

### 2.5. Runtime Measurement

Runtime was reported as decomposition latency per epoch. For each method, we timed the decomposition for each 2 s segment, aggregated timings across epochs, and reported the mean decomposition time per segment.

### 2.6. Inter-Method Comparison Protocol

Inter-method comparisons used the bin-wise Opt1 configuration for each method within each (kind, NSR bin). For each epoch in a given bin, we computed RRMSE for all methods, converted values to within-epoch ranks (1 = lowest RRMSE), and summarised the rank distribution as P1|P2|P3|P4 along with the mean rank. PCC-based ranks were computed analogously and reported as supplementary confirmation.

## 3. Results

### 3.1. Within-Method Hyperparameter Behaviour and Selections

#### 3.1.1. VMD

**Figure 4:**
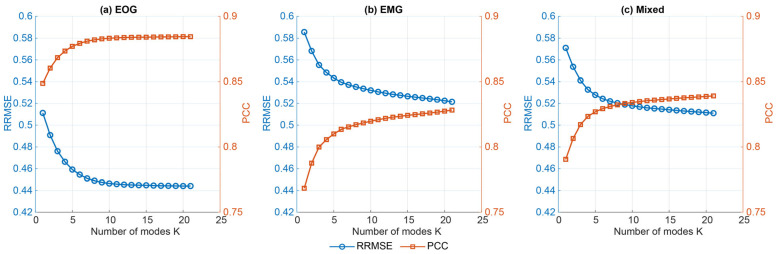
Mean ± SD oracle reconstruction performance of VMD across full hyperparameter grid, pooled across all NSR [−10, 10] dB bin for EOG, EMG, and mixed segments. Left *y*-axis: RRMSE (lower is better), right *y*-axis: PCC (higher is better).

Pooling across NSR, VMD oracle reconstructions improved monotonically as the number of modes K increased, with a near-linear increase in decomposition time. For EOG segments (N = 1250), mean RRMSE decreased from 0.51 ± 0.12 at K = 4 to 0.44 ± 0.13 at K = 24, with corresponding PCC gains. Similar trends were observed for EMG-only and mixed contamination (Figure 4).

**Figure 5:**
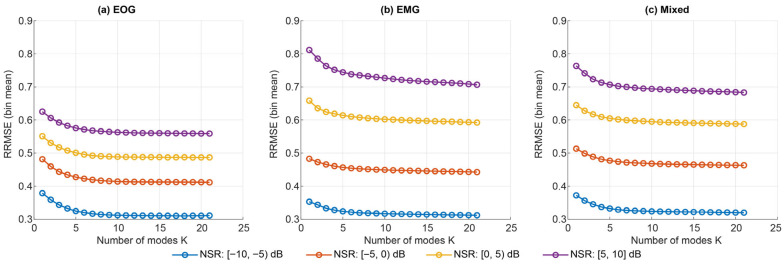
NSR-stratified oracle performance for VMD across EOG, EMG, and mixed contamination.

When stratifying by NSR bins [−10, −5), [−5, 0), [0, 5), [5, 10] (and ALL [−10, 10]), the Best-by-RRMSE configurations consistently favoured high mode counts (approximately K ≈ 21–24) across EOG, EMG, and mixed regimes (Figure 5). Reconstruction error increased with contamination level; however, the Best region remained in the high-K range Table 2.

Very small mode counts (K < 7) consistently produced substantially higher reconstruction errors and were excluded from subsequent analysis of Opt2 for clarity.

**Figure 6:**
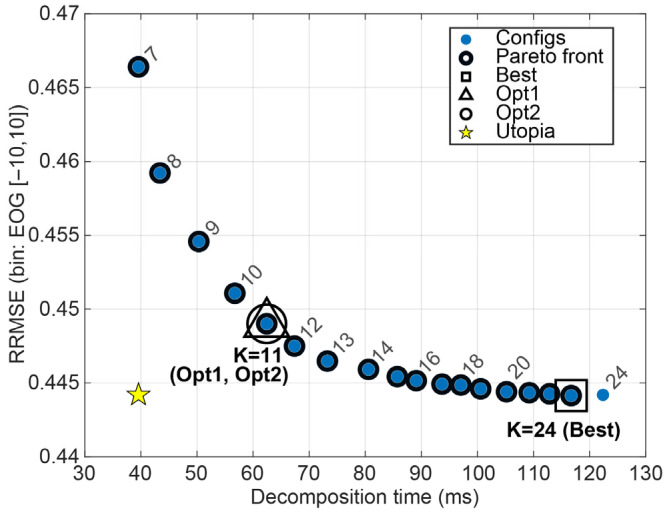
Performance–latency landscape for VMD configurations, showing the trade-off between mean decomposition time per 2 s epoch and mean oracle RRMSE. Highlighted markers denote the selected Best, Opt1, and Opt2 configurations for EOG noise kind.

Under the effect-size criterion (Opt1; |d|≤0.05), VMD selected moderate K values that were statistically indistinguishable from Best, yet substantially faster. Opt2 selections lay near the knee of the performance–latency curve and closely tracked Opt1 for EOG contamination, while selecting a slightly more aggressive speed–quality compromise for EMG and mixed regimes (Figure 6). Overall, VMD achieves its lowest oracle errors at high K, whereas moderate K (≈11–13) provides a practical operating point with only a small RRMSE increase but roughly half the decomposition time under our implementation. The bin-wise Best, Opt1, and Opt2 hyperparameters for VMD are summarised in Table 2.

#### 3.1.2. SSA

**Figure 7:**
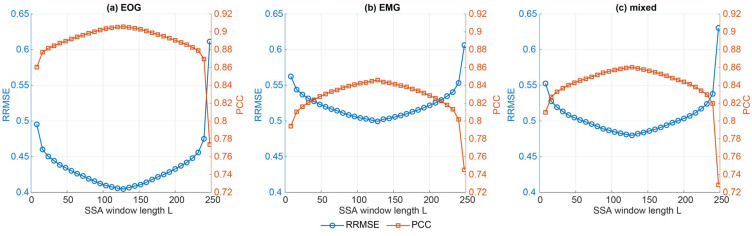
Mean ± SD oracle reconstruction performance of SSA across full hyperparameter grid, pooled all NSR [−10, 10] dB bin for EOG, EMG, and mixed segments. Left *y*-axis: RRMSE (lower is better), right *y*-axis: PCC (higher is better).

Across window lengths L = 8…248, SSA exhibited a pronounced convex dependence of oracle reconstruction quality on L, with Best performance consistently occurring at moderate windows near L≈128 (Figure 7). In the ALL bin [−10,10] dB, the Best-by-RRMSE configuration was L = 128 for EOG, EMG, and mixed contamination.

**Figure 8:**
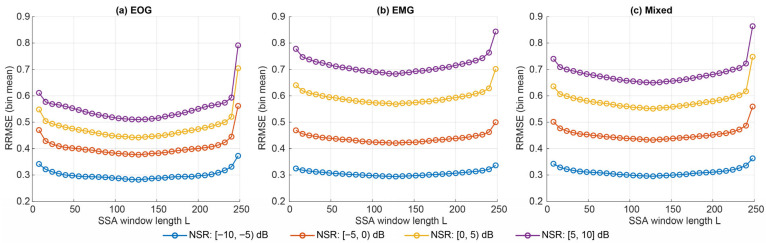
NSR-stratified oracle performance for SSA across EOG, EMG, and mixed contamination.

When stratifying into NSR bins [−10,−5), [−5,0), [0,5), [5,10], the Best SSA window lengths remained stable, clustering within the L≈120–136 range across noise kinds (Figure 8, Table 3). As expected, reconstruction error increased with contamination level, but the near-optimal SSA region was largely unchanged across bins.

**Figure 9:**
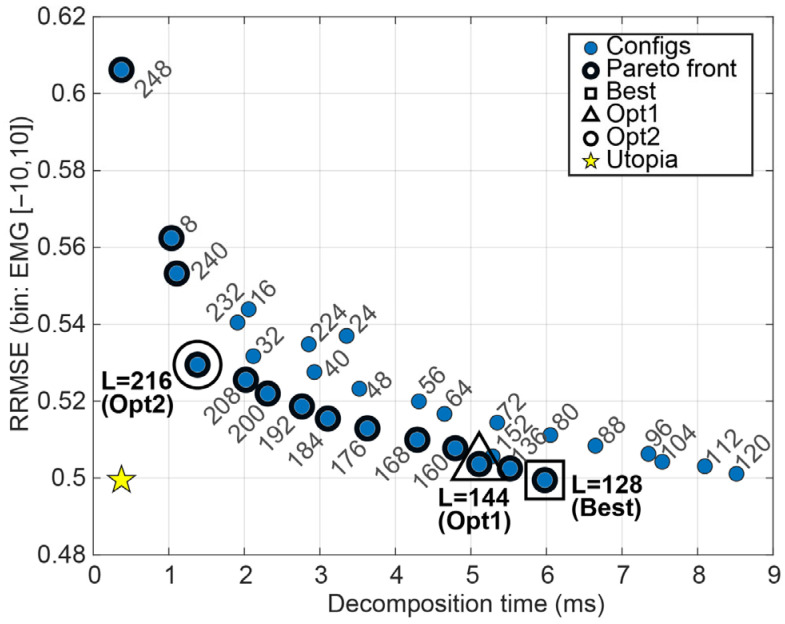
Performance–latency landscape for SSA configurations, showing the trade-off between mean decomposition time per 2 s epoch and mean oracle RRMSE. Highlighted markers denote the selected Best, Opt1, and Opt2 configurations for EMG noise kind.

Under the effect-size criterion (|d|≤0.05 vs. Best), Opt1 selected L = 144 globally across kinds, yielding near-Best reconstruction quality with a modest latency reduction. In contrast, the Pareto-based Opt2 criterion selected a much larger window (L = 216), providing a substantial speed-up (Figure 9). The SSA decomposition time showed a non-monotonic dependence on L, with a peak near L≈120 and lower runtimes at both small and large windows; this reflects the changing trajectory–matrix shape, with SVD cost typically highest when the matrix is closest to square (here around T/2) and lower for more rectangular cases. Overall, SSA’s near-optimal window length was highly stable, while Opt2 reflects an explicit performance–latency compromise rather than a near-equivalent alternative to Best. The bin-wise Best, Opt1, and Opt2 hyperparameters for SSA are summarised in Table 3.

#### 3.1.3. DWT

**Figure 10:**
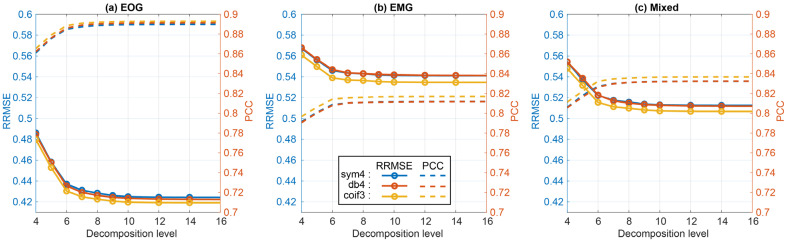
Mean ± SD oracle reconstruction performance of DWT across full hyperparameter grid, pooled across all NSR [−10, 10] dB bin for EOG, EMG, and mixed segments. Left *y*-axis: RRMSE (lower is better), right *y*-axis: PCC (higher is better).

We evaluated three mother wavelets (sym4, db4, coif3) and decomposition levels J∈{4,5,6,7,8,9,10,12,14,16} (Figure 10). Oracle reconstruction quality varied with both wavelet and depth, with coif3 consistently achieving the lowest mean RRMSE at higher levels. Decomposition time increased with level but remained in the millisecond range across the explored grid.

**Figure 11:**
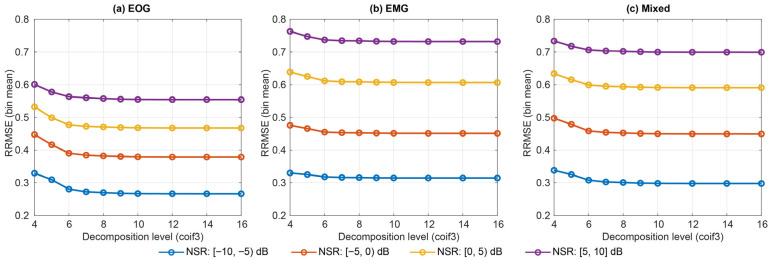
NSR-stratified oracle performance for DWT (wavelet: coif3) across EOG, EMG, and mixed contamination.

Under NSR stratification [−10, −5), [−5, 0), [0, 5), [5, 10] (and ALL [−10, 10]), the Best-by-RRMSE DWT setting was remarkably stable: coif3, J = 16 was selected for EOG, EMG, and mixed contamination in every bin (Figure 11, Table 4). The reconstruction error increased with the contamination level.

**Figure 12:**
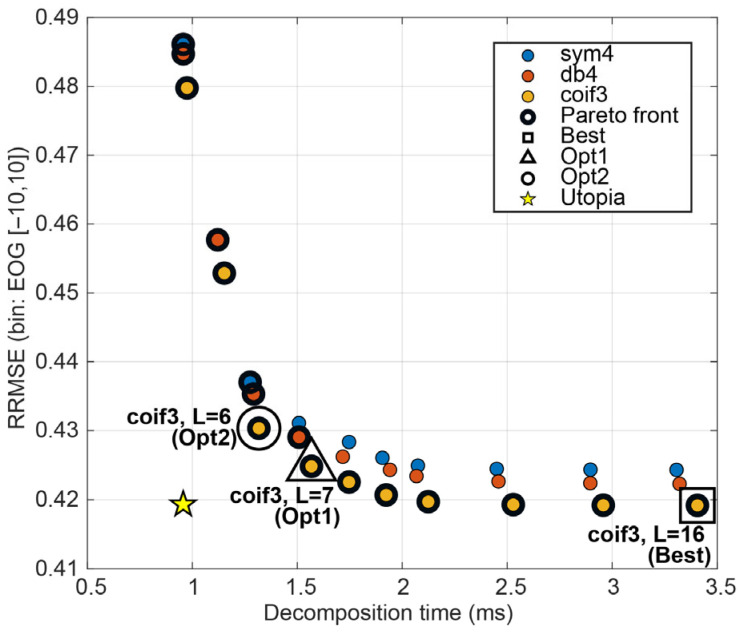
Performance–latency landscape for DWT configurations, showing the trade-off between mean decomposition time per 2 s epoch and mean oracle RRMSE. Highlighted markers denote the selected Best, Opt1, and Opt2 configurations for EOG noise kind.

Under the effect-size criterion (Opt1; |d|≤0.05 vs. Best), DWT consistently selected moderate depths (typically J ≈ 6–7) that were statistically indistinguishable from the Best reconstructions, while slightly reducing runtime. Opt2 selections closely matched this behaviour, reflecting that DWT’s performance–latency curve is already favourable. Modest reductions in level yield small latency gains with only minor changes in oracle reconstruction metrics (Figure 12). Overall, DWT provides a stable near-optimal operating region (coif3 with moderate-to-high depth), with Opt1/Opt2 favouring shallower levels for a lightweight speed benefit. The bin-wise Best, Opt1, and Opt2 hyperparameters for DWT are summarised in Table 4.

To provide an illustrative bridge from oracle recoverability to a deployable pipeline within the wavelet family, we evaluated DWT universal soft-threshold shrinkage (depth L = 6; detail levels 1–6 thresholded) using three mother wavelets (sym4, db4, coif3) on the same validation subset. Table 5 reports bin-wise performance for ocular-only mixtures (N = 1250). In the mildest ocular bin (−10,−5] dB, db4 achieved the lowest mean RRMSE (0.375 vs. 0.381 for sym4 and 0.392 for coif3), and paired testing showed coif3 was slightly worse than db4 (median ΔRRMSE = +0.0169 for coif3–db4, Holm-adjusted $p\;=\;1.33\times 10^{-4}$; Supplementary Table S4). In contrast, for (−5,0], (0,5], and (5,10] dB, coif3 consistently achieved the lowest mean RRMSE (0.456, 0.560, and 0.718, respectively) and its advantage increased with contamination severity; for example, in the highest bin (5,10] dB, coif3 outperformed sym4 with a median ΔRRMSE = −0.1813 (win rate 0.879; Holm-adjusted $p\;=\;6.87\times 10^{-45}$; Supplementary Table S4). Across all ocular epochs (ALL-bin), coif3 remained best overall (mean RRMSE 0.534 vs. 0.628 for sym4 and 0.693 for db4). Collectively, these results indicate that, within a method family with established practical selectors, oracle-guided wavelet preferences can largely translate under standard coefficient thresholding. Full paired statistical comparisons (median differences, win rates, and Holm-adjusted *p*-values for all wavelet pairs per bin) are provided in Supplementary Table S4.

#### 3.1.4. CEEMDAN

**Figure 13:**
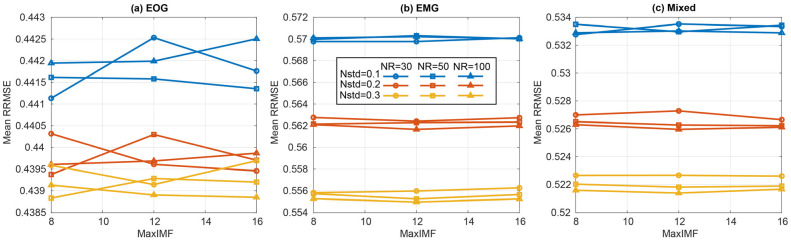
Mean ± SD oracle reconstruction performance of CEEMDAN across full hyperparameter grid, pooled across all NSR [−10, 10] dB bin for EOG, EMG, and mixed segments. *y*-axis represents RRMSE (lower is better).

Across the CEEMDAN grid (noise amplitude Nstd, ensemble size NR, and maximum IMFs MaxIMF), oracle reconstruction quality varied only modestly, with the most visible separation driven by Nstd rather than NR or MaxIMF (Figure 13). In contrast, decomposition time was dominated by NR, with NR = 100 consistently far slower than NR = 30 for similar RRMSE/PCC.

**Figure 14:**
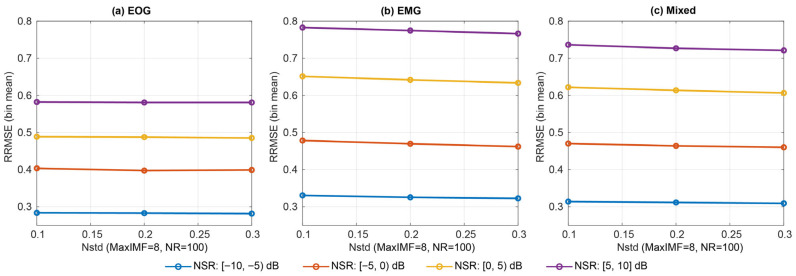
NSR-stratified oracle performance for CEEMDAN (MaxIMF = 8, NR = 100) across EOG, EMG, and mixed contamination.

Under NSR stratification [−10, −5), [−5, 0), [0, 5), [5, 10] (and ALL [−10, 10]), the Best-by-RRMSE selections were most often in the high-noise regime (Nstd = 0.3) across EOG, EMG, and mixed contamination (Figure 14, Table 6). However, Best sometimes relied on larger NR (e.g., NR = 50 for EOG, NR = 100 for EMG/mixed in the ALL bin), producing substantially higher latency for only marginal gains in reconstruction metrics.

**Figure 15:**
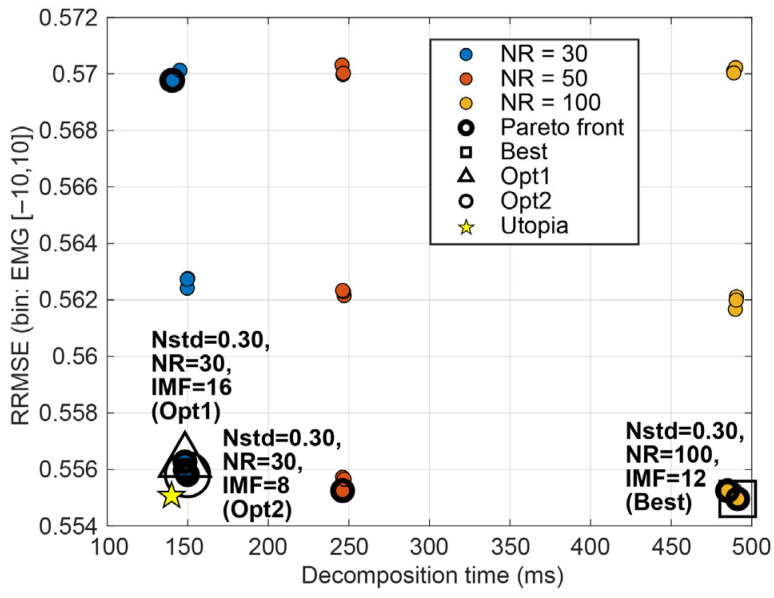
Performance–latency landscape for CEEMDAN configurations, showing the trade-off between mean decomposition time per 2 s epoch and mean oracle RRMSE. Highlighted markers denote the selected Best, Opt1, and Opt2 configurations for EMG noise kind.

Under the effect-size criterion (Opt1; |d|≤0.05 vs. Best), CEEMDAN consistently moved to the lowest ensemble size (NR = 30) (typically retaining Nstd = 0.3 for EMG/mixed), achieving large latency reductions while remaining essentially indistinguishable from Best in RRMSE/PCC. Opt2 showed the same pattern, selecting NR = 30 as the most practical point on the performance–latency front, with MaxIMF shifting modestly (often 12) but with limited impact relative to NR (Figure 15). Overall, CEEMDAN’s primary practical determinant is ensemble size: while Best-by-RRMSE may favour larger NR, both Opt1 and Opt2 converge to NR = 30 as a near-optimal operating region with markedly lower runtime. The bin-wise Best, Opt1, and Opt2 hyperparameters for CEEMDAN are summarised in Table 6.

**Table 2:** Bin-wise VMD hyperparameter selections and oracle performance summary. $\bar{t}$ represents the decomposition time in seconds (s).

Kind	Bin	N	BEST				Opt1				Opt2			
			K	RRMSE	PCC	$\bar{t}$	K	RRMSE	PCC	$\bar{t}$	K	RRMSE	PCC	$\bar{t}$
EOG	[−10, −5)	303	21	0.310 ± 0.062	0.949 ± 0.021	0.109	12	0.313 ± 0.061	0.948 ± 0.021	0.067	11	0.314 ± 0.061	0.948 ± 0.021	0.06
	[−5, 0)	310	24	0.411 ± 0.087	0.907 ± 0.039	0.122	12	0.415 ± 0.086	0.906 ± 0.038	0.067	11	0.417 ± 0.085	0.905 ± 0.038	0.06
	[0, 5)	314	22	0.487 ± 0.106	0.866 ± 0.059	0.113	11	0.491 ± 0.103	0.864 ± 0.059	0.062	11	0.491 ± 0.103	0.864 ± 0.059	0.06
	[5, 10]	323	23	0.559 ± 0.100	0.821 ± 0.069	0.117	13	0.563 ± 0.099	0.818 ± 0.069	0.073	12	0.564 ± 0.098	0.818 ± 0.069	0.07
	[−10, 10]	1250	23	0.444 ± 0.129	0.885 ± 0.069	0.117	11	0.449 ± 0.129	0.882 ± 0.070	0.062	11	0.449 ± 0.129	0.882 ± 0.070	0.06
EMG	[−10, −5)	281	24	0.312 ± 0.050	0.949 ± 0.017	0.122	18	0.314 ± 0.049	0.948 ± 0.017	0.097	12	0.318 ± 0.049	0.947 ± 0.017	0.07
	[−5, 0)	312	24	0.443 ± 0.080	0.893 ± 0.040	0.122	17	0.446 ± 0.080	0.891 ± 0.041	0.094	12	0.450 ± 0.080	0.889 ± 0.041	0.07
	[0, 5)	333	24	0.592 ± 0.099	0.797 ± 0.075	0.122	19	0.596 ± 0.098	0.794 ± 0.076	0.101	12	0.604 ± 0.098	0.788 ± 0.077	0.07
	[5, 10]	324	24	0.707 ± 0.097	0.694 ± 0.101	0.122	22	0.711 ± 0.096	0.690 ± 0.101	0.113	13	0.727 ± 0.093	0.673 ± 0.102	0.07
	[−10, 10]	1250	24	0.522 ± 0.170	0.828 ± 0.118	0.122	15	0.529 ± 0.173	0.822 ± 0.122	0.086	12	0.534 ± 0.175	0.818 ± 0.125	0.07
Mixed	[−10, −5)	530	24	0.320 ± 0.057	0.946 ± 0.020	0.122	16	0.322 ± 0.056	0.945 ± 0.020	0.089	11	0.326 ± 0.056	0.944 ± 0.020	0.06
	[−5, 0)	474	24	0.463 ± 0.076	0.883 ± 0.041	0.122	15	0.467 ± 0.076	0.881 ± 0.042	0.086	11	0.470 ± 0.075	0.879 ± 0.042	0.06
	[0, 5)	497	24	0.588 ± 0.083	0.803 ± 0.063	0.122	17	0.592 ± 0.083	0.800 ± 0.063	0.094	12	0.597 ± 0.082	0.797 ± 0.063	0.07
	[5, 10]	499	24	0.683 ± 0.088	0.720 ± 0.089	0.122	19	0.687 ± 0.088	0.716 ± 0.090	0.101	12	0.695 ± 0.089	0.708 ± 0.092	0.07
	[−10, 10]	2000	24	0.511 ± 0.158	0.839 ± 0.104	0.122	12	0.519 ± 0.160	0.833 ± 0.108	0.067	12	0.519 ± 0.160	0.833 ± 0.108	0.07

**Table 3:** Bin-wise SSA hyperparameter selections and oracle performance summary. $\bar{t}$ represents the decomposition time in seconds (s).

Kind	Bin	N	BEST				Opt1				Opt2			
			L	RRMSE	PCC	$\bar{t}$	L	RRMSE	PCC	$\bar{t}$	L	RRMSE	PCC	$\bar{t}$
EOG	[−10, −5)	303	128	0.282 ± 0.066	0.958 ± 0.020	0.006	136	0.284 ± 0.066	0.957 ± 0.020	0.006	216	0.303 ± 0.065	0.951 ± 0.021	0.001
	[−5, 0)	310	128	0.377 ± 0.087	0.923 ± 0.035	0.006	136	0.379 ± 0.087	0.922 ± 0.036	0.006	216	0.407 ± 0.087	0.909 ± 0.039	0.001
	[0, 5)	314	128	0.442 ± 0.106	0.891 ± 0.054	0.006	144	0.446 ± 0.108	0.889 ± 0.055	0.005	216	0.485 ± 0.104	0.867 ± 0.060	0.001
	[5, 10]	323	128	0.510 ± 0.101	0.854 ± 0.063	0.006	144	0.513 ± 0.101	0.852 ± 0.064	0.005	216	0.564 ± 0.100	0.818 ± 0.070	0.001
	[−10, 10]	1250	128	0.405 ± 0.125	0.906 ± 0.060	0.006	144	0.409 ± 0.124	0.904 ± 0.061	0.005	216	0.442 ± 0.132	0.885 ± 0.071	0.001
EMG	[−10, −5)	281	128	0.294 ± 0.047	0.955 ± 0.015	0.006	136	0.296 ± 0.048	0.955 ± 0.015	0.006	200	0.307 ± 0.048	0.951 ± 0.016	0.002
	[−5, 0)	312	128	0.421 ± 0.072	0.905 ± 0.034	0.006	144	0.424 ± 0.073	0.903 ± 0.035	0.005	216	0.444 ± 0.076	0.893 ± 0.038	0.001
	[0, 5)	333	128	0.568 ± 0.088	0.817 ± 0.064	0.006	136	0.572 ± 0.089	0.814 ± 0.066	0.006	216	0.602 ± 0.092	0.791 ± 0.073	0.001
	[5, 10]	324	128	0.683 ± 0.085	0.723 ± 0.083	0.006	136	0.687 ± 0.085	0.719 ± 0.083	0.006	216	0.727 ± 0.088	0.676 ± 0.097	0.001
	[−10, 10]	1250	128	0.500 ± 0.163	0.846 ± 0.104	0.006	144	0.504 ± 0.165	0.843 ± 0.107	0.005	216	0.529 ± 0.174	0.822 ± 0.122	0.001
Mixed	[−10, −5)	530	128	0.295 ± 0.055	0.955 ± 0.018	0.006	136	0.297 ± 0.055	0.954 ± 0.018	0.006	216	0.316 ± 0.057	0.948 ± 0.020	0.001
	[−5, 0)	474	128	0.432 ± 0.073	0.900 ± 0.036	0.006	136	0.435 ± 0.073	0.899 ± 0.036	0.006	216	0.458 ± 0.076	0.886 ± 0.040	0.001
	[0, 5)	497	128	0.552 ± 0.082	0.830 ± 0.056	0.006	136	0.555 ± 0.082	0.828 ± 0.056	0.006	216	0.589 ± 0.084	0.803 ± 0.063	0.001
	[5, 10]	499	128	0.650 ± 0.094	0.752 ± 0.083	0.006	136	0.651 ± 0.094	0.751 ± 0.084	0.006	216	0.692 ± 0.089	0.712 ± 0.092	0.001
	[−10, 10]	2000	128	0.480 ± 0.155	0.860 ± 0.094	0.006	144	0.484 ± 0.156	0.858 ± 0.095	0.005	216	0.511 ± 0.163	0.838 ± 0.108	0.001

**Table 4:** Bin-wise DWT hyperparameter selections and oracle performance summary. * a/b represents wavelet/level. $\bar{t}$ represents the decomposition time in seconds (s).

Kind	Bin	N	BEST				Opt1				Opt2			
			a/b *	RRMSE	PCC	$\bar{t}$	a/b *	RRMSE	PCC	$\bar{t}$	a/b *	RRMSE	PCC	$\bar{t}$
EOG	[−10, −5)	303	coif3/16	0.266 ± 0.071	0.961 ± 0.020	0.003	coif3/8	0.270 ± 0.070	0.961 ± 0.020	0.002	db4/7	0.274 ± 0.069	0.959 ± 0.021	0.002
	[−5, 0)	310	coif3/16	0.379 ± 0.094	0.920 ± 0.039	0.003	coif3/8	0.382 ± 0.092	0.919 ± 0.039	0.002	coif3/6	0.390 ± 0.090	0.916 ± 0.039	0.001
	[0, 5)	314	coif3/16	0.468 ± 0.112	0.875 ± 0.059	0.003	coif3/7	0.473 ± 0.110	0.873 ± 0.059	0.002	coif3/6	0.477 ± 0.110	0.870 ± 0.060	0.001
	[5, 10]	323	coif3/16	0.554 ± 0.112	0.821 ± 0.080	0.003	coif3/8	0.558 ± 0.112	0.819 ± 0.080	0.002	coif3/6	0.564 ± 0.111	0.815 ± 0.080	0.001
	[−10, 10]	1250	coif3/16	0.419 ± 0.145	0.893 ± 0.076	0.003	coif3/7	0.425 ± 0.144	0.891 ± 0.076	0.002	coif3/6	0.430 ± 0.143	0.889 ± 0.077	0.001
EMG	[−10, −5)	281	coif3/16	0.315 ± 0.047	0.948 ± 0.016	0.003	coif3/7	0.316 ± 0.047	0.948 ± 0.016	0.002	coif3/6	0.318 ± 0.047	0.947 ± 0.016	0.001
	[−5, 0)	312	coif3/16	0.451 ± 0.075	0.888 ± 0.039	0.003	coif3/7	0.453 ± 0.075	0.888 ± 0.039	0.002	coif3/6	0.455 ± 0.075	0.887 ± 0.039	0.001
	[0, 5)	333	coif3/16	0.607 ± 0.094	0.786 ± 0.075	0.003	coif3/7	0.609 ± 0.094	0.784 ± 0.076	0.002	coif3/6	0.612 ± 0.094	0.782 ± 0.076	0.001
	[5, 10]	324	coif3/16	0.732 ± 0.093	0.667 ± 0.102	0.003	coif3/7	0.735 ± 0.093	0.665 ± 0.103	0.002	coif3/6	0.737 ± 0.093	0.662 ± 0.103	0.001
	[−10, 10]	1250	coif3/16	0.535 ± 0.175	0.817 ± 0.126	0.003	coif3/6	0.539 ± 0.176	0.814 ± 0.128	0.001	coif3/6	0.539 ± 0.176	0.814 ± 0.128	0.001
Mixed	[−10, −5)	530	coif3/16	0.298 ± 0.061	0.953 ± 0.019	0.003	coif3/8	0.301 ± 0.061	0.952 ± 0.019	0.002	db4/7	0.304 ± 0.061	0.951 ± 0.020	0.002
	[−5, 0)	474	coif3/16	0.449 ± 0.077	0.889 ± 0.041	0.003	coif3/8	0.452 ± 0.077	0.888 ± 0.041	0.002	coif3/6	0.458 ± 0.077	0.885 ± 0.041	0.001
	[0, 5)	497	coif3/16	0.591 ± 0.087	0.799 ± 0.066	0.003	coif3/7	0.595 ± 0.087	0.797 ± 0.066	0.002	coif3/6	0.599 ± 0.087	0.793 ± 0.067	0.001
	[5, 10]	499	coif3/16	0.699 ± 0.092	0.702 ± 0.095	0.003	coif3/7	0.703 ± 0.091	0.699 ± 0.095	0.002	coif3/6	0.706 ± 0.091	0.696 ± 0.096	0.001
	[−10, 10]	2000	coif3/16	0.507 ± 0.173	0.837 ± 0.114	0.003	coif3/7	0.511 ± 0.172	0.835 ± 0.114	0.002	coif3/6	0.515 ± 0.171	0.832 ± 0.115	0.001

**Table 5:** Bin-wise mean RRMSE and mean PCC for DWT universal soft-threshold shrinkage (depth L=6; detail levels 1–6 thresholded), comparing mother wavelets sym4, db4, and coif3 on the ocular-only validation subset. Bins are defined by ocular NSR $Z_{oc}$ (dB): [−10,−5), [−5,0), [0,5), (5,10], and ALL. N denotes the number of epochs in each bin. Lower RRMSE and higher PCC are better.

Ocular NSR Bin	N	sym4 RRMSE	sym4 PCC	db4 RRMSE	db4 PCC	coif3 RRMSE	coif3 PCC	Best (RRMSE)
[−10, −5)	303	0.3811	0.9235	0.3753	0.9267	0.3916	0.9191	db4
[−5, 0)	310	0.5038	0.8700	0.5182	0.8670	0.4559	0.8879	coif3
[0, 5)	314	0.6741	0.7905	0.7395	0.7680	0.5604	0.8333	coif3
[5, 10]	323	0.9358	0.6737	1.1148	0.6195	0.7184	0.7526	coif3
[−10, 10]	1250	0.6284	0.8144	0.6933	0.7953	0.5344	0.8482	coif3

**Table 6:** Bin-wise CEEMDAN hyperparameter selections and oracle performance summary. * a/b/c represents Nstd/NR/MaxIMF. $\bar{t}$ represents the decomposition time in seconds (s).

Kind	Bin	N	BEST				Opt1				Opt2			
			a/b/c *	RRMSE	PCC	$\bar{t}$	a/b/c *	RRMSE	PCC	$\bar{t}$	a/b/c *	RRMSE	PCC	$\bar{t}$
EOG	[−10, −5)	303	0.30/50/8	0.280 ± 0.071	0.958 ± 0.021	0.246	0.10/30/8	0.282 ± 0.069	0.957 ± 0.021	0.14	0.30/30/12	0.281 ± 0.068	0.958 ± 0.021	0.148
	[−5, 0)	310	0.20/50/16	0.397 ± 0.090	0.913 ± 0.039	0.246	0.10/30/8	0.401 ± 0.090	0.912 ± 0.039	0.14	0.20/30/16	0.397 ± 0.087	0.914 ± 0.038	0.15
	[0, 5)	314	0.30/100/8	0.485 ± 0.108	0.866 ± 0.062	0.489	0.10/30/8	0.488 ± 0.109	0.865 ± 0.063	0.14	0.30/30/12	0.486 ± 0.109	0.866 ± 0.062	0.148
	[5, 10]	323	0.30/100/16	0.580 ± 0.110	0.805 ± 0.080	0.485	0.10/30/8	0.584 ± 0.111	0.801 ± 0.084	0.14	0.20/30/16	0.581 ± 0.111	0.804 ± 0.081	0.15
	[−10, 10]	1250	0.30/50/8	0.439 ± 0.147	0.884 ± 0.080	0.246	0.10/30/8	0.441 ± 0.147	0.882 ± 0.082	0.14	0.30/30/12	0.439 ± 0.147	0.884 ± 0.080	0.148
EMG	[−10, −5)	281	0.30/100/12	0.322 ± 0.047	0.946 ± 0.016	0.491	0.30/30/16	0.322 ± 0.048	0.946 ± 0.017	0.148	0.30/30/12	0.322 ± 0.048	0.946 ± 0.017	0.148
	[−5, 0)	312	0.30/100/12	0.462 ± 0.075	0.883 ± 0.040	0.491	0.30/30/16	0.464 ± 0.074	0.882 ± 0.040	0.148	0.30/30/8	0.463 ± 0.074	0.883 ± 0.040	0.15
	[0, 5)	333	0.30/100/12	0.633 ± 0.092	0.765 ± 0.079	0.491	0.30/30/16	0.635 ± 0.092	0.763 ± 0.080	0.148	0.30/30/8	0.635 ± 0.092	0.764 ± 0.080	0.15
	[5, 10]	324	0.30/100/16	0.766 ± 0.085	0.629 ± 0.104	0.485	0.30/30/16	0.768 ± 0.084	0.627 ± 0.103	0.148	0.30/30/12	0.767 ± 0.084	0.629 ± 0.104	0.148
	[−10, 10]	1250	0.30/100/12	0.555 ± 0.183	0.800 ± 0.139	0.491	0.30/30/16	0.556 ± 0.184	0.799 ± 0.140	0.148	0.30/30/8	0.556 ± 0.183	0.799 ± 0.139	0.15
Mixed	[−10, −5)	530	0.30/50/12	0.309 ± 0.061	0.949 ± 0.020	0.246	0.30/30/16	0.310 ± 0.061	0.949 ± 0.020	0.148	0.30/30/8	0.310 ± 0.061	0.949 ± 0.020	0.15
	[−5, 0)	474	0.30/100/12	0.460 ± 0.077	0.884 ± 0.042	0.491	0.30/30/16	0.461 ± 0.076	0.884 ± 0.041	0.148	0.30/30/12	0.460 ± 0.077	0.884 ± 0.042	0.148
	[0, 5)	497	0.30/100/12	0.606 ± 0.088	0.788 ± 0.070	0.491	0.30/30/16	0.608 ± 0.088	0.787 ± 0.070	0.148	0.30/30/16	0.608 ± 0.088	0.787 ± 0.070	0.148
	[5, 10]	499	0.30/100/12	0.720 ± 0.094	0.680 ± 0.103	0.491	0.30/30/16	0.722 ± 0.095	0.678 ± 0.104	0.148	0.30/30/16	0.722 ± 0.095	0.678 ± 0.104	0.148
	[−10, 10]	2000	0.30/100/12	0.521 ± 0.176	0.827 ± 0.122	0.491	0.30/30/16	0.523 ± 0.176	0.826 ± 0.123	0.148	0.30/30/16	0.523 ± 0.176	0.826 ± 0.123	0.148

### 3.2. Inter-Method Comparison Under Bin-Wise Opt1 Tuning

We compared VMD, SSA, DWT, and CEEMDAN under bin-wise Opt1 tuning. For each contamination type (EOG, EMG, mixed) and each NSR bin (four bins plus the pooled ALL range), we ranked methods within each epoch by RRMSE and summarised the resulting rank distributions using percentiles (P1–P4; Table 7) and mean rank.

Across the 15 conditions (3 kinds×(4 NSR bins+ALL)), SSA ranked first in 14/15 conditions, reflecting the most stable Opt1 performance across bins. The only exception was mild EOG contamination [−10,−5), where DWT most frequently ranked first and VMD most frequently ranked last. For EOG, SSA’s relative advantage increased with NSR: near parity with DWT in [−5,0), followed by clearer separation in [0,5) and [5,10]. Over pooled EOG [−10,10], SSA remained first overall, with DWT typically second.

Under EMG contamination, SSA most frequently ranked first in every bin, while CEEMDAN most frequently ranked last, with the separation increasing at higher NSR. VMD most often ranked second and DWT third. For mixed contamination, SSA ranked first across all bins, while the runner-up depended on regime: DWT more often ranked second at lower mixed NSR, whereas VMD more often became second at higher mixed NSR.

**Figure 16:**
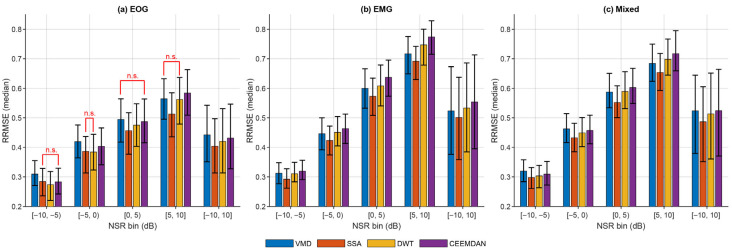
Bin-wise comparison of Opt1-tuned VMD, SSA, DWT, and CEEMDAN using oracle reconstructions. Panels show EOG, EMG, and mixed contamination. Within each panel, bars report the median RRMSE for each method within each NSR bin ([−10, −5), [−5, 0), [0, 5), [5, 10], and ALL = [−10, 10]), with whiskers indicating the interquartile range (25th–75th percentiles). Brackets (annotated with n.s. representing not significant inference) denote method pairs that were not significantly different within that bin under paired Wilcoxon signed-rank testing with multiple-comparison adjustment (*p* ≥ 0.05).

PCC-based rank distributions closely mirrored the RRMSE ordering (Supplementary Table S1). Exploratory paired Wilcoxon signed-rank tests (multiple-comparison adjusted) found many pairwise contrasts significant (Supplementary Tables S2 and S3). Bin-wise RRMSE distributions are shown in Figure 16.

**Table 7:** Bin-wise inter-method comparison of Opt1-tuned VMD, SSA, DWT, and CEEMDAN using oracle reconstructions. For each contamination kind (EOG, EMG, mixed) and NSR bin ([−10, −5), [−5, 0), [0, 5), [5, 10], and ALL = [−10, 10]), methods were ranked per epoch by RRMSE (lower is better). Each cell reports the rank distribution as P1|P2|P3|P4, i.e., the percentage of epochs for which the method ranked 1st–4th, followed by the mean rank in parentheses (smaller is better). N denotes the number of epochs contributing to the bin.

Kind	Bin	N	Rank Distribution Percentiles and Mean Rank: P1|P2|P3|P4 (Mean Rank)			
			VMD	SSA	DWT	CEEMDAN
EOG	[−10, −5)	303	1.7|4.6|20.1|73.6 (3.66)	27.4|30.0|33.3|9.2 (2.24)	44.9|32.0|20.5|2.6 (1.81)	26.1|33.3|26.1|14.5 (2.29)
	[−5, 0)	310	2.9|15.2|34.8|47.1 (3.26)	40.6|31.9|15.8|11.6 (1.98)	38.4|31.3|21.6|8.7 (2.01)	18.1|21.6|27.7|32.6 (2.75)
	[0, 5)	314	6.1|21.7|37.9|34.4 (3.01)	53.2|26.1|13.4|7.3 (1.75)	24.8|30.6|26.4|18.2 (2.38)	15.9|21.7|22.3|40.1 (2.87)
	[5, 10]	323	5.3|34.7|41.5|18.6 (2.73)	67.8|21.1|7.7|3.4 (1.47)	20.7|30.3|25.1|23.8 (2.52)	6.2|13.9|25.7|54.2 (3.28)
	[−10, 10]	1250	3.9|18.2|33.3|44.6 (3.18)	47.0|28.5|17.4|7.1 (1.85)	31.6|30.1|24.8|13.5 (2.20)	17.4|23.2|24.6|34.8 (2.77)
EMG	[−10, −5)	281	2.5|54.4|28.8|14.2 (2.55)	86.1|11.0|2.5|0.4 (1.17)	8.9|19.9|39.9|31.3 (2.94)	2.5|14.6|28.8|54.1 (3.35)
	[−5, 0)	312	9.9|55.1|23.1|11.9 (2.37)	78.8|17.3|2.9|1.0 (1.26)	9.0|17.6|42.6|30.8 (2.95)	2.2|9.9|31.4|56.4 (3.42)
	[0, 5)	333	21.3|48.0|24.9|5.7 (2.15)	67.0|22.8|8.4|1.8 (1.45)	8.7|21.6|49.5|20.1 (2.81)	3.0|7.5|17.1|72.4 (3.59)
	[5, 10]	324	23.5|54.6|18.2|3.7 (2.02)	69.8|21.9|7.1|1.2 (1.40)	6.2|17.3|59.0|17.6 (2.88)	0.6|6.2|15.7|77.5 (3.70)
	[−10, 10]	1250	10.8|54.2|25.4|9.7 (2.34)	79.0|15.7|4.6|0.7 (1.27)	7.8|19.4|45.4|27.4 (2.92)	2.5|10.7|24.6|62.2 (3.47)
Mixed	[−10, −5)	530	2.5|17.4|27.5|52.6 (3.30)	56.4|24.5|16.0|3.0 (1.66)	28.9|32.1|25.3|13.8 (2.24)	12.3|26.0|31.1|30.6 (2.80)
	[−5, 0)	474	5.9|20.3|32.9|40.9 (3.09)	61.8|22.2|11.0|5.1 (1.59)	21.3|30.2|30.4|18.1 (2.45)	11.0|27.4|25.7|35.9 (2.86)
	[0, 5)	497	8.5|43.5|30.0|18.1 (2.58)	75.7|15.1|6.2|3.0 (1.37)	10.3|25.6|36.8|27.4 (2.81)	5.6|15.9|27.0|51.5 (3.24)
	[5, 10]	499	13.0|51.7|28.7|6.6 (2.29)	77.2|17.4|4.6|0.8 (1.29)	7.6|19.6|42.3|30.5 (2.96)	2.2|11.2|24.4|62.1 (3.46)
	[−10, 10]	2000	5.1|29.8|31.4|33.7 (2.94)	68.3|19.4|9.5|2.8 (1.47)	18.1|28.2|32.2|21.6 (2.57)	8.5|22.6|27.0|42.0 (3.02)

## 4. Discussion

Our results provide a decomposition-focused view of four commonly used single-channel families (DWT, CEEMDAN, VMD, SSA) under the oracle reconstruction setting and can be summarised in relation to the practical questions, as mentioned previously in the Introduction.

RQ1 (hyperparameter stability across regimes). Across the NSR bins and contamination kinds considered, SSA and DWT showed comparatively stable near-optimal regions under the explored grids. SSA’s Best settings clustered around a moderate window length (near L≈128). DWT’s Best settings consistently favoured coif3 at the highest tested depth, while Opt1/Opt2 selected shallower levels with only small changes in oracle metrics.

For VMD and CEEMDAN, the Opt1 selections showed clearer regime dependence by contamination type. Under bin-wise Opt1 tuning, EOG segments selected relatively modest VMD mode counts (K = 11–13 across bins), whereas EMG and mixed segments selected higher values (K≈15–22 for EMG and K≈12–19 for mixed), with the highest-NSR bins favouring the largest K in both cases. For CEEMDAN (Nstd/NR/MaxIMF), Opt1 was stable within each contamination kind but shifted across kinds: EOG bins consistently selected 0.10/30/8, while EMG and mixed bins consistently selected 0.30/30/16 under the tested grid. These patterns suggest that reasonable operating points may be more transferable for some families (SSA, DWT) than others, and that preferred settings can depend on contamination regime.

RQ2 (regime-dependent competitiveness across families). Under bin-wise Opt1 tuning, SSA was most frequently top-ranked by epoch-wise RRMSE across most conditions, particularly under EMG and mixed contamination. The clearest exception occurred in the lowest EOG NSR bin, where DWT was often most competitive and rank distributions overlapped more strongly between methods. Overall, these results suggest that relative rankings can vary with both artifact type and contamination level, with some regimes showing clearer separation and others appearing closer to “tied” behaviour under the oracle/Opt1 lens.

RQ3 (performance–runtime trade-offs and diminishing returns). The within-method sweeps illustrate that improved oracle performance can coincide with higher computational cost; however, the degree of this trade-off differs by family. VMD benefited from increasing K in terms of oracle metrics but with near-linear time growth, making moderate K values a plausible compromise under Opt1/Opt2. CEEMDAN’s runtime was dominated by ensemble size (NR) in our implementation; larger ensembles often provided only modest oracle gains within the tested grid. By contrast, DWT and SSA were comparatively inexpensive per epoch in our MATLAB implementation; their Opt1/Opt2 operating points tended to retain most of the Best performance while reducing depth/complexity.

Notably, oracle reconstruction errors were generally higher under EMG and mixed regimes than under EOG regimes in this benchmark; the Opt1 selections for VMD and CEEMDAN tended to require higher decomposition capacity in EMG/mixed than in EOG. While this does not imply that EMG is universally harder in all settings, it is consistent with the idea that EMG-heavy regimes can be more demanding for single-channel decomposition-based suppression under the contamination model and hyperparameter ranges considered here.

### 4.1. Practical Considerations Beyond Performance

Our primary inter-method comparison is performance-driven (epoch-wise rank distributions under bin-wise Opt1 tuning using oracle reconstructions). In applied settings, however, method choice is also influenced by latency constraints, the transparency of decomposition outputs, and the practical burden of tuning and component handling.

Runtime practicality. In our MATLAB setup (MATLAB R2023b, i7 CPU, 32 GB RAM), DWT and SSA executed in the millisecond range per 2 s epoch, VMD was slower and increased with the number of modes, and CEEMDAN was slowest (≈0.14–0.49 s per epoch under our implementation). Because absolute timings depend on software optimisation and hardware, these results are best interpreted as relative indicators within a consistent implementation rather than fixed deployment estimates.

Decision practicality (component-handling burden). Decomposition-based denoising requires translating components into a reconstruction decision. DWT yields a structured set of sub-bands that can support simple attenuation rules. VMD produces K modes which can remain manageable at moderate K, while larger K may introduce redundancy and increase handling burden. CEEMDAN produces multiple IMFs and can be sensitive to noise-assistance settings. SSA can yield many candidate components; however, in practice, it is often paired with grouping rules rather than purely manual component-by-component curation. These considerations are most relevant in regimes where oracle performance is closely overlapping between methods.

Tuning burden and stability. Under our explored grids, SSA and DWT showed relatively broad near-optimal regions across NSR bins, suggesting that once a reasonable configuration is established, extensive retuning may be less critical within this benchmark. By comparison, VMD’s K-dependent trade-offs and CEEMDAN’s interacting parameters (particularly ensemble size vs. runtime) make tuning choices more consequential, especially when latency constraints are present.

Implications for method choice under this evaluation. Under oracle/Opt1 conditions, SSA most frequently ranked first across most bins and contamination types, while DWT was most competitive in the lowest EOG NSR bin. In regimes where rank distributions overlap (i.e., methods appear effectively tied), practical considerations, such as latency and component-handling complexity, may reasonably guide method choice. These observations should be interpreted as patterns within our synthetic contamination and oracle reconstruction framework, rather than as deployment-ready prescriptions.

### 4.2. Interpreting Oracle Results and Implications

#### 4.2.1. Interpreting Oracle Performance

Oracle reconstructions are defined using the clean reference and, therefore, serve as a best-case reference point for what is recoverable from a given decomposition representation under the tested contamination regimes. They do not constitute a deployable method without an explicit selection or weighting rule. Within this framing, the strong oracle/Opt1 performance of SSA, particularly under EMG and mixed contamination, suggests that SSA representations can support low-error reconstructions when component weights are chosen optimally. The EOG results were more regime-dependent, with closer competition at low EOG NSR and clearer separation at higher EOG NSR, consistent with the idea that relative advantages can vary with contamination level in single-channel settings. The oracle should therefore be interpreted as a best-case reference within a fixed component basis, not as evidence that the decomposition itself yields perfectly separated neural and artifact components.

#### 4.2.2. From Oracle to Deployment: The Selection Gap

A practical pipeline requires an explicit mechanism to approximate the oracle’s component decisions. Candidate approaches include lightweight heuristics (e.g., component bandpower ratios, time–frequency concentration measures, or transient morphology indicators) or learned selectors that output per-component attenuation weights. Quantifying the resulting gap-to-oracle across contamination regimes helps to distinguish decompositions that are not only recoverable in principle but also tractable in practice.

To provide an empirical bridge in a setting where established selector rules exist, we added a practical baseline within the wavelet family using standard coefficient thresholding (DWT, universal soft thresholding). Under this baseline, coif3 achieved the best overall performance on ocular mixtures and was consistently best in moderate-to-high ocular regimes ($Z_{oc}>-5$ dB; Table 5), broadly aligning with the oracle-guided preference for coif3 under nontrivial contamination. In the mildest ocular bin (−10,−5] dB, db4 slightly outperformed coif3, and paired testing confirmed that this small difference is statistically detectable (Supplementary Table S4). Together, these results emphasize that oracle analysis quantifies representational headroom, whereas realized performance depends on selector behaviour and operating regime.

From a deployment perspective, the results suggest that decomposition choice and hyperparameter selection should be matched to both the contamination regime and the latency budget. The Opt1 operating points are useful when maximizing recoverability is the primary goal, whereas the Opt2 points provide a more pragmatic choice when runtime or energy constraints are important. In this sense, the benchmark is not only comparative but also prescriptive: it indicates which settings are likely to offer the best accuracy–efficiency trade-off before any selector is designed. More broadly, practical selector design is likely to remain family-specific: wavelet pipelines naturally align with thresholding rules, VMD with mode-selection or mode-weighting criteria based on frequency content and bandwidth, SSA with grouping or subspace-selection rules, and EMD-family methods with IMF-selection heuristics. Likewise, within-family extensions, such as adaptive or successive VMD variants, stationary or dual-tree complex wavelet transforms, and other advanced decomposition variants, may further improve practical performance; however, evaluating these would shift the emphasis from inter-family upper-bound comparison to within-family method development. We therefore view the present study as a decomposition-centric reference point that can guide these next-stage selector and family-extension studies in a more controlled and interpretable way.

#### 4.2.3. Limitations

A key limitation of the present benchmark is that the oracle reconstruction requires access to the clean target and, therefore, cannot be used directly in deployment. Importantly, the oracle does not assume that the decomposition has already achieved perfect separation of neural and artifact components; rather, it computes the best achievable reconstruction within the span of the obtained component set under bounded weighting. This makes it a decomposition-centric upper bound rather than a practical denoiser. Relatedly, the benchmark relies on synthetic mixtures because the question posed here, how much clean EEG is recoverable from a given decomposition under ideal weighting, requires a known clean reference. In this sense, the use of semi-simulated data is intentional: the clean EEG and artifact exemplars are real signals drawn from EEGdenoiseNet, while the simplification lies in the additive mixing model and the controlled NSR design. We therefore interpret the results as upper-bound recoverability under controlled conditions, while recognising that genuine EEG artifacts can be more nonlinear, nonstationary, and context-dependent than the mixtures considered here. Real-data validation remains important; however, in this study, it is most naturally framed as a next step for evaluating practical selectors rather than as a substitute for the oracle benchmark itself. Furthermore, because epochs are not linked to subject/session identifiers, epoch-wise inferential testing may overstate certainty if samples are correlated; accordingly, we emphasise rank distributions and robust descriptive summaries and treat inferential tests as exploratory. Runtime comparisons are implementation-dependent and should be interpreted comparatively within our setup.

#### 4.2.4. Future Work

Immediate next steps are (i) evaluating heuristic and learned selectors to approximate oracle weighting and reporting the resulting gap-to-oracle across regimes; and (ii) assessing task preservation (e.g., ERP morphology, SSVEP peaks, and downstream decoding stability) to check that improvements in reconstruction metrics do not come at the cost of attenuating neurophysiologically meaningful content. More complex adaptive pipelines (e.g., regime-aware switching across methods) could then be explored once baseline selector performance is established.

## 5. Conclusions

This work presented an oracle-based benchmark of four commonly used one-dimensional decomposition families (VMD, SSA, DWT, and CEEMDAN) for single-channel EEG artifact suppression under controlled EOG, EMG, and mixed contamination across NSR bins [−10,−5),[−5,0),[0,5),[5,10] and the pooled range [−10,10]. By computing an oracle reconstruction through the constrained re-weighting of decomposition components against a clean reference, we used a decomposition-focused reference point to examine how recoverability varies with both noise regime and decomposition granularity.

Under bin-wise Opt1 tuning (effect-size–aware “good-enough” settings), SSA was the most consistently competitive method, ranking first in 14/15 contamination conditions. In the pooled [−10,10] bin, SSA achieved the best mean ranks for EOG (1.85), EMG (1.27), and mixed (1.47) contamination. The main exception was mild EOG contamination [−10,−5), where DWT most frequently ranked first, suggesting that relative performance can be regime-dependent even under consistent tuning rules.

Beyond oracle performance, we summarised practical considerations that may influence method choice in applied pipelines, including runtime, tuning burden, and component-handling complexity. While absolute timings are implementation-dependent, DWT and SSA were comparatively lightweight in our MATLAB setup, VMD offered adjustable granularity with higher compute cost, and CEEMDAN’s runtime was substantially higher under the tested settings.

Because oracle weighting uses access to the clean reference, these findings should be interpreted as an upper bound on what each decomposition can recover under the tested regimes. Translating this bound into deployable pipelines requires practical component selection/weighting; future work should quantify the gap to oracle and validate task preservation (e.g., ERP/SSVEP integrity) on broader real-world and wearable artifacts.

## Figures and Tables

**Table 1:** Number of synthetic 2 s epochs per contamination kind (EOG-only, EMG-only, mixed) after stratification into NSR bins [−10,−5), [−5,0), [0,5), [5,10] and the pooled range [−10,10].

Bin	EOG-Only	EMG-Only	Mixed
[−10, −5)	303	281	530
[−5, 0)	310	312	474
[0, 5)	314	333	497
[5, 10]	323	324	499
[−10, 10]	1250	1250	2000

## Data Availability

The code supporting the findings of this study is publicly available at GitHub: https://github.com/usmanqamarshaikh/oracle-eeg-recoverability-benchmark (accessed on 26 December 2025).

## Supplementary Materials

The following supporting information can be downloaded at: https://www.mdpi.com/article/10.3390/s26092581/s1, Table S1: RankDist Opt1 PCC; Table S2: Pairwise Opt1 Signrank; Table S3: Pairwise Opt1 Signrank; Table S4: PracticalWavelet.
